# Supplementary material for: Time-series transcriptome analysis identified differentially expressed genes in broiler chicken infected with mixed Eimeria species
Source: Front Genet. 2022 Aug 8;13:886781. doi: 10.3389/fgene.2022.886781 (PMC9393255; doi:10.3389/fgene.2022.886781)
Supplement: Supplementary file 2 [file DataSheet1.ZIP › 4dpi_KEGG.Gsea.1625100298584/KEGG_VALINE_LEUCINE_AND_ISOLEUCINE_BIOSYNTHESIS.html]

Details for gene set KEGG\_VALINE\_LEUCINE\_AND\_ISOLEUCINE\_BIOSYNTHESIS[GSEA]

|  || Dataset | TMM\_4dpi\_gct\_format\_4dpi\_gct\_format.Class\_4dpi.cls #PC\_versus\_NC.Class\_4dpi.cls #PC\_versus\_NC\_repos |
| Phenotype | Class\_4dpi.cls#PC\_versus\_NC\_repos |
| Upregulated in class | 0 |
| GeneSet | KEGG\_VALINE\_LEUCINE\_AND\_ISOLEUCINE\_BIOSYNTHESIS |
| Enrichment Score (ES) | -0.6837723 |
| Normalized Enrichment Score (NES) | -1.3461721 |
| Nominal p-value | 0.14530988 |
| FDR q-value | 0.3858068 |
| FWER p-Value | 0.9998 |
Table: GSEA Results Summary

  

Fig 1: Enrichment plot: KEGG\_VALINE\_LEUCINE\_AND\_ISOLEUCINE\_BIOSYNTHESIS      
 Profile of the Running ES Score & Positions of GeneSet Members on the Rank Ordered List

  

| SYMBOL | TITLE | RANK IN GENE LIST | RANK METRIC SCORE | RUNNING ES | CORE ENRICHMENT || 1 | PDHA2 | na | 6524 | -0.049 | -0.5088 | No |
| 2 | PDHB | na | 8626 | -0.242 | -0.5127 | Yes |
| 3 | LARS2 | na | 8678 | -0.248 | -0.3422 | Yes |
| 4 | IARS2 | na | 9805 | -0.387 | -0.1632 | Yes |
| 5 | BCAT1 | na | 10469 | -0.491 | 0.1284 | Yes |
Table: GSEA details [plain text format]

  

Fig 2: KEGG\_VALINE\_LEUCINE\_AND\_ISOLEUCINE\_BIOSYNTHESIS      
 Blue-Pink O' Gram in the Space of the Analyzed GeneSet

  

Fig 3: KEGG\_VALINE\_LEUCINE\_AND\_ISOLEUCINE\_BIOSYNTHESIS: Random ES distribution      
 Gene set null distribution of ES for **KEGG\_VALINE\_LEUCINE\_AND\_ISOLEUCINE\_BIOSYNTHESIS**
